# An Unsupported Preference for Intravenous Antibiotics

**DOI:** 10.1371/journal.pmed.1001825

**Published:** 2015-05-19

**Authors:** Ho Kwong Li, Ambrose Agweyu, Mike English, Philip Bejon

**Affiliations:** 1 Bone Infection Unit, Nuffield Orthopaedic Centre, Oxford University Hospitals, Oxford, United Kingdom; 2 Kenya Medical Research Institute Wellcome Trust Research Programme, Nairobi, Kenya; 3 Centre for Tropical Medicine and Global Health, Nuffield Department of Medicine, University of Oxford, Oxford, United Kingdom; 4 Kenya Medical Research Institute Wellcome Trust Research Programme, Kilifi, Kenya

## Abstract

Philip Bejon and colleagues reflect on the widespread belief in the superiority of intravenous antibiotics.

**Summary Points**

- Antibiotics that are well absorbed after oral administration are available, and the best current evidence suggests they are safe and effective for many conditions.
- Belief in the superiority of intravenous antibiotics is widespread among health professionals and patients, but it is not supported by good evidence. Expanding the evidence base will provide patients and clinicians with further reassurance in specific situations, but reasons for the belief in the strength of intravenous therapy also need to be understood and addressed.
- Trials expanding the evidence base might follow noninferiority designs, based on the precedent of widespread intravenous use. For many indications, the theoretical reasons for preferring intravenous therapy are not strong, and the risks of intravenous therapy are well established. It would be more logical for many indications to regard oral antibiotics as the default position and require trial designs to test the superiority of intravenous therapy.
- Clarity regarding the harms and benefits of intravenous antibiotics is needed. There is potential to change global clinical practice for the better, reducing health care costs and minimizing harm to patients.

## Intravenous Antibiotic Use

Antibiotics given intravenously are commonly used in both high- and low-income countries. Available evidence from well-established antibiotic stewardship programmes in high-income settings suggests this is frequently unnecessary [1,2]. Data from low-income settings, though limited, demonstrate similar findings. An audit of therapeutic interventions in children with uncomplicated pneumonia in a Peruvian hospital found that 100% (*n* = 42) of subjects could have been treated with oral antibiotics [3]. Our own experience of admissions to paediatric wards in Kenya is that many children receive intravenous penicillin and large numbers of patients with malaria receive parenteral therapy despite clinical features suggesting that oral medication would be appropriate.

Intravenous therapy may result in harmful complications such as phlebitis, extravasation injury, thrombosis, and local or systemic infection including bacteraemia. Intravenous therapy also prolongs the duration of inpatient stay, causing pain and inconvenience to the patient and financial cost to the health care system. The risk of bacteraemia in peripheral intravenous, peripherally inserted central, and central venous catheters can be as high as 0.1%, 2.4%, and 4.4%, respectively [4]. For higher-income countries, the combined risk is 0.2–2 bloodstream infections per 1,000 intravenous catheter days [5]. A significant risk of hospital-acquired infection has also been observed in lower-income countries [6]. The cost of prolonged inpatient stay for intravenous antibiotics is estimated at £4,500 per day. This expense and inconvenience can be reduced by outpatient intravenous antibiotic administration, but even outpatient intravenous treatment comes at a significant cost estimated at £1,800 via outpatient administration. Furthermore, in selecting an antibiotic suitable for once-daily dosing, clinicians may need to choose unnecessarily broad-spectrum agents [7].

## Why Is Intravenous Therapy Needed?

An absolute requirement for parenteral antibiotics is present when patients cannot swallow or absorb oral antibiotics (for instance, during critical illness) or when intolerances or microbial susceptibility requires an agent that is effective if given intravenously but that would have poor oral bioavailability. These indications (e.g., meningitis and intensive care unit [ICU] admission) probably justify only a minority of current intravenous antibiotic prescriptions [1,2].

The more rapidly achieved peak antibiotic levels after intravenous dosing may be important when treating rapidly progressing infections such as severe sepsis and bacterial meningitis, as reflected in treatment guidelines and in the “surviving sepsis campaign” [8], although it has been shown that an early switch to oral antibiotics is safe in selected meningitis patients [9]. However, many clinicians and patients may infer from these guidelines that intravenous antibiotics are generally “stronger,” leading clinicians and patients to prefer intravenous antibiotics for other conditions. This belief may be further reinforced by guidelines that promote prereferral, injectable antibiotics for children with a “danger sign,” although many of them may be able to take oral medication [10], or by guidelines suggesting that prognostic signs associated with severe pneumonia in adults indicate the need for intravenous therapy [11,12]. The message implied is that serious illnesses require strong intravenous antibiotics.

Aside from rapidly achieving peak levels, is there any other theoretical advantage of intravenous therapy? For the most commonly used antibiotics such as beta-lactams, glycopeptides, or macrolides, antimicrobial killing is not dependent on the peak levels but rather on the period of time during which antibiotic levels are above the minimum inhibitory concentration [13]. Even allowing for the higher doses that can be given intravenously, the time above minimum inhibitory concentration is similar for well-absorbed oral antibiotics compared with intravenous antibiotics. Exceptions to this rule are aminoglycosides and quinolones, in which antimicrobial killing is related to the peak concentrations achieved (however, aminoglycosides are not orally absorbed and quinolones are not used extensively by the intravenous route, so there is no obvious oral versus intravenous comparison within these classes). Well-absorbed oral antibiotics, such as amoxicillin, clindamycin and doxycycline, are available, with bioavailability at >75%, 90%, and >95%, respectively [14–16]. Many of these antibiotics achieve adequate concentrations in tissues such as prostate tissue or bone. The high cerebrospinal fluid (CSF) concentrations required for treating neurosyphillis may not be achieved with oral penicillins but may be achievable with other oral agents for which there are limited data on efficacy. Intravenous regimes might be thought to ensure adherence to treatment, although this could also be achieved by directly observed oral therapy. There is little theoretical basis for believing that intravenous antibiotics are simply “better” or “stronger” than oral antibiotics.

## Randomized Controlled Trials

No large single randomized controlled trial (RCT) has demonstrated superiority in intravenous treatment, but a number of trials (many adopting the strategy of early oral switch) have demonstrated equivalence of oral versus intravenous antibiotic therapy, as illustrated by the following references.

**Fig 1:**
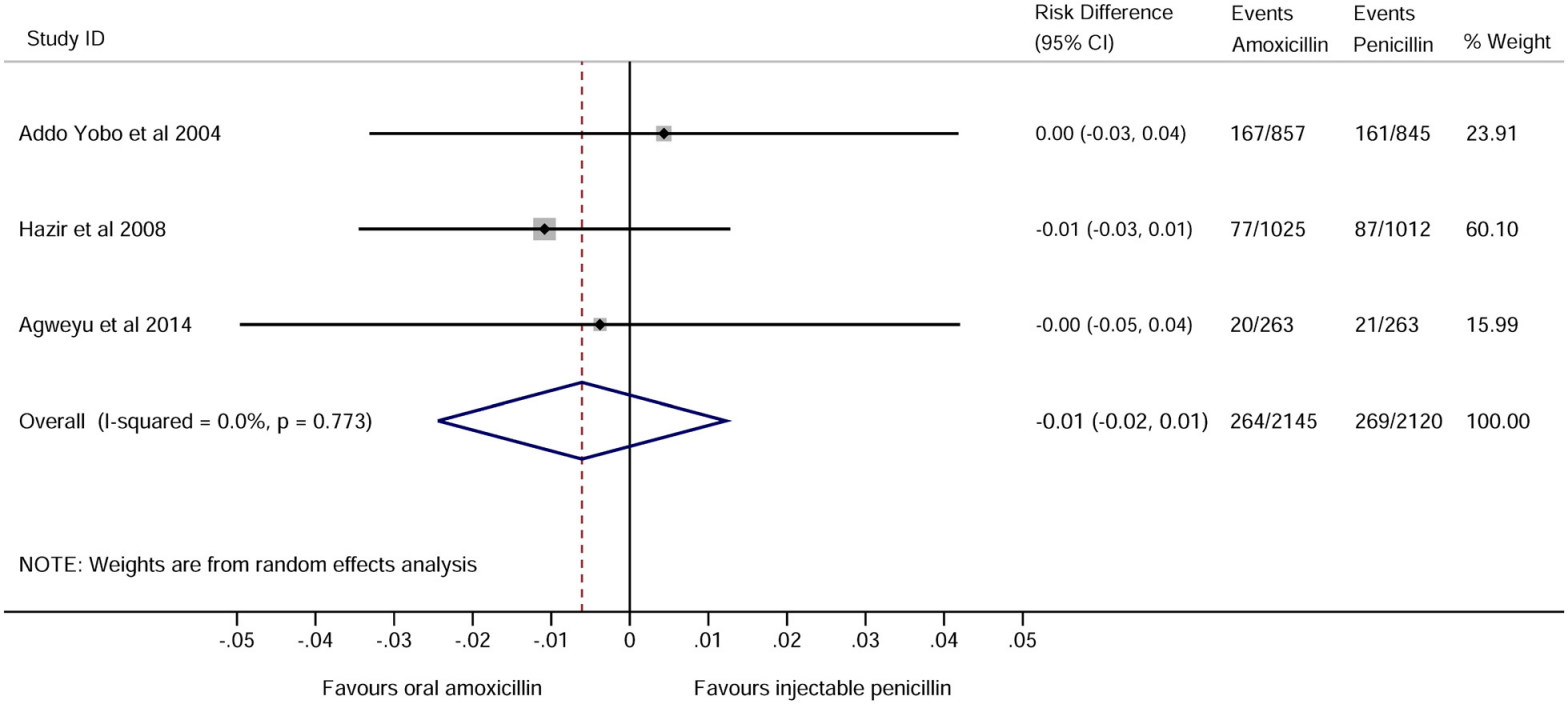
Forest plot for trials comparing treatment failure among children in low-income settings who were treated with amoxicillin or benzyl penicillin for severe pneumonia. See supplementary figure 2 of [19].

For childhood pneumonia, the leading cause of hospitalization and death in children globally, noninferiority of oral amoxicillin versus injectable penicillin has been studied in three large trials conducted in low-income countries [17–19] and one small study undertaken in the United Kingdom [20]. When pooled, these trials include over 4,000 children recruited across nine countries and show no difference in clinical treatment failure rates (pooled risk difference -0.01 95% CI -0.02 to 0.01, shown in Fig 1, which was taken from supplementary figure 2 of [19] using the search criteria as applied in S1 Fig in August 2014). The World Health Organisation guidelines have recently been revised to allow outpatient treatment with oral antibiotics for moderately severe pneumonia [10].

RCTs in adults with severe and nonsevere pneumonia include comparisons of oral versus intravenous antibiotics or comparisons of early switches from intravenous to oral antibiotics versus full intravenous courses. These RCTs have not shown any advantage of intravenous treatment, including studies in the United States [21], the Netherlands [22], Spain [23], and Germany [24].

For acute pyelonephritis, six paediatric RCTs (*n* = 917) concluded that an early switch to oral antibiotics is equivalent to longer intravenous regimes [25]. In patients with “low risk” febrile neutropenia (i.e., haemodynamically stable, without organ failure or an obvious source of severe infection), 22 RCTs (*n* = 2,372) concluded that oral treatment (or early switches to oral treatment) is an acceptable alternative to intravenous treatment [26].

One RCT demonstrated equivalent outcomes for oral antibiotics and intravenous antibiotics in infective endocarditis, recruiting 85 intravenous drug users (IVDUs) with uncomplicated right-sided native valve *Staphylococcus aureus* endocarditis [27]. Five RCTs (*n* = 205) were included in a meta-analysis comparing oral regimes to either intravenous antibiotics alone or intravenous antibiotics with an oral combination in chronic osteomyelitis. No statistically significant difference was detected in remission rates between the intravenous and the oral treatment groups [28]. In acute paediatric osteomyelitis, two retrospective cohort studies, one comparing early oral switch to intravenous antibiotic regimes (*n* = 1,969) and the other a direct oral versus intravenous comparison (*n* = 2,060), both reported that oral therapy was not associated with higher risk of treatment failures [29,30].

Ongoing trials examining the equivalence of oral and intravenous antibiotics include trials in neonatal sepsis in low-income settings [31] and in bone and joint infection in high-income settings [32]. Demonstration of equivalence in these trials would further reduce the need for hospitalization among neonates with suspected sepsis and among adults with bone or joint infection.

## A Preference for Intravenous Therapy

The preference for intravenous therapy may be a legacy of the discovery of antibiotics. Penicillin acquired enduring fame by providing a dramatic cure following intravenous administration to acutely unwell patients [33]. Antibiotics that were well absorbed when administered orally became available in the years that followed but made less of an impression without similar stories of “miracle cures.”

Salvarsan was the first antibiotic “magic bullet” to be routinely used and became the preferred treatment for syphilis. It required repeated parenteral injections, leading one US doctor to famously remark that “even the poor can scarcely be expected to submit with good grace to repeated barbarities offered in the name of medicine” [34]. This remark betrays some of the economic prejudice of the era but may also overlook medical history: dramatic and intrusive treatments are often preferred by patients and doctors, an effect that may contribute to a placebo effect [35]. It is therefore possible that “repeated barbarities” in fact influence some patients and their doctors to prefer intravenous therapy over the less impressive tablets on offer.

When randomized controlled trials are designed to demonstrate “equivalence,” this implies that our default position should be to regard intravenous therapy as “safe and proven” and that we require substantial evidence to reassure us before changing practice. In fact, for many indications the theoretical reasons for preferring intravenous therapy are not strong, and the risks of intravenous therapy are well established. It would be more logical for many indications to regard oral antibiotics as the default position and require trial designs to test the superiority of intravenous therapy.

Initial doses of intravenous therapy in acutely life-threatening conditions may be needed to provide immediate effective antimicrobial plasma concentrations. In cases in which intravenous access is required for supportive care (such as fluids), there seems little harm in giving intravenous antibiotics. There is little merit in trials to compare oral therapy with intravenous therapy for the first dose when admission to hospital for supportive care is required in any case, but when hospital admission or parenteral therapy in the community might be avoided with oral therapy, there can be substantial savings to be made and risks avoided for the patient and the health system [17]. In cases in which initial doses must be intravenous, an early switch to oral antibiotics is usually justified. It is not logical to make a switch to oral antibiotics dependent on “response to therapy”; this is a contingency that erroneously implies that intravenous therapy is somehow “stronger.”

In order to consolidate the evidence base, large RCTs should now test higher-risk indications for intravenous antibiotics such as bacteraemia, meningitis, and bone infections. The risk of oral antibiotics in these trials can be mitigated by giving the first few doses intravenously. The complexity of selecting antibiotic agents and a broad range of conditions to consider makes large, pragmatic trials of strategy an attractive design.

However, even for conditions covered by the current evidence base, substantial resistance to the use of oral antibiotics is encountered. Resistance to change comes from clinician reinforcement behaviour and other cognitive biases, fear of litigation, and reimbursement strategies adopted by insurance companies in which hospitalisation can be most readily justified by intravenous antibiotic use when oral therapy would have been sufficient [22,23,36]. The preference for intravenous antibiotics needs challenging and only large-scale trial data can overcome strongly held personal anecdotes and third-party institutional policy assumptions in which hospitalisation equals intravenous therapy. Medicine is indeed repeatedly barbarous when unnecessary iatrogenic harm is done.

## Supporting Information

S1 Fig Search criteria and studies for meta-analysis of treatment for severe pneumonia. (TIFF)

## Abbreviations

CSF: cerebrospinal fluid
ICU: intensive care unit
IVDU: intravenous drug user
RCT: randomized controlled trial

## References

[1] Boyles TH, Whitelaw A, Bamford C, Moodley M, Bonorchis K, Morris V, et al Antibiotic stewardship ward rounds and a dedicated prescription chart reduce antibiotic consumption and pharmacy costs without affecting inpatient mortality or re-admission rates. PLoS One. 2013;8: e79747 10.1371/journal.pone.0079747 24348995 PMC3857167

[2] Dellit TH, Owens RC, McGowan JE Jr., Gerding DN, Weinstein RA, Burke JP, et al Infectious Diseases Society of America and the Society for Healthcare Epidemiology of America guidelines for developing an institutional program to enhance antimicrobial stewardship. Clin Infect Dis. 2007;44: 159–177. 17173212 10.1086/510393

[3] Carreazo NY, Bada CA, Chalco JP, Huicho. Audit of therapeutic interventions in inpatient children using two scores: are they evidence-based in developing countries? BMC Health Serv Res. 2004;4: 40 15625006 10.1186/1472-6963-4-40 PMC544399

[4] Maki DG, Kluger DM, Crnich CJ. The risk of bloodstream infection in adults with different intravascular devices: a systematic review of 200 published prospective studies. Mayo Clin Proc. 2006;81: 1159–1171. 16970212 10.4065/81.9.1159

[5] Edgeworth J. Intravascular catheter infections. J Hosp Infect. 2009;73: 323–330. 10.1016/j.jhin.2009.05.008 19699555

[6] Aiken AM, Mturi N, Njuguna P, Mohammed S, Berkley JA, Mwangi I, et al Risk and causes of paediatric hospital-acquired bacteraemia in Kilifi District Hospital, Kenya: a prospective cohort study. Lancet. 2011;378: 2021–2027. 10.1016/S0140-6736(11)61622-X 22133536 PMC3242162

[7] Chapman AL, Dixon S, Andrews D, Lillie PJ, Bazaz R, Patchett JD. Clinical efficacy and cost-effectiveness of outpatient parenteral antibiotic therapy (OPAT): a UK perspective. J Antimicrob Chemother. 2009;64: 1316–1324. 10.1093/jac/dkp343 19767623

[8] Levy MM, Artigas A, Phillips GS, Rhodes A, Beale R, Osborn T, et al Outcomes of the Surviving Sepsis Campaign in intensive care units in the USA and Europe: a prospective cohort study. Lancet Infect Dis. 2012;12: 919–924. 10.1016/S1473-3099(12)70239-6 23103175

[9] Karageorgopoulos DE, Valkimadi PE, Kapaskelis A, Rafailidis PI, Falagas ME. Short versus long duration of antibiotic therapy for bacterial meningitis: a meta-analysis of randomised controlled trials in children. Arch Dis Child. 2009;94: 607–614. 10.1136/adc.2008.151563 19628879

[10] World-Health-Organisation. Pocket book of hospital care for children: guidelines for the management of common illnesses with limited resources. Geneva: World-Health-Organisation; 2013. 24006557

[11] Lim WS, Baudouin SV, George RC, Hill AT, Jamieson C, Le Jeune I, et al BTS guidelines for the management of community acquired pneumonia in adults: update 2009. Thorax 64 2009; Suppl.3: iii1–55. 10.1136/thx.2009.121434 19783532

[12] Mandell LA, Wunderink RG, Anzueto A, Bartlett JG, Campbell GD, Dean NC, et al Infectious Diseases Society of America/American Thoracic Society consensus guidelines on the management of community-acquired pneumonia in adults. Clin Infect Dis 44 2007; Suppl.2: S27–72. 17278083 10.1086/511159 PMC7107997

[13] Vogelman B, Gudmundsson S, Leggett J, Turnidge J, Ebert S, Craig WA. Correlation of antimicrobial pharmacokinetic parameters with therapeutic efficacy in an animal model. J Infect Dis. 1988;158: 831–847. 3139779 10.1093/infdis/158.4.831

[14] Saivin S, Houin G. Clinical pharmacokinetics of doxycycline and minocycline. Clin Pharmacokinet. 1988;15: 355–366. 3072140 10.2165/00003088-198815060-00001

[15] Bouazza N, Pestre V, Jullien V, Curis E, Urien S, Salmon D, et al Population pharmacokinetics of clindamycin orally and intravenously administered in patients with osteomyelitis. Br J Clin Pharmacol. 2012;74: 971–977. 10.1111/j.1365-2125.2012.04292.x 22486719 PMC3522810

[16] Arancibia A, Guttmann J, Gonzalez G, Gonzalez C. Absorption and disposition kinetics of amoxicillin in normal human subjects. Antimicrob Agents Chemother. 1980;17: 199–202. 7387142 10.1128/aac.17.2.199 PMC283758

[17] Hazir T, Fox LM, Nisar YB, Fox MP, Ashraf YP, MacLeod WB, et al Ambulatory short-course high-dose oral amoxicillin for treatment of severe pneumonia in children: a randomised equivalency trial. Lancet. 2008;371: 49–56. 10.1016/S0140-6736(08)60071-9 18177775

[18] Addo-Yobo E, Chisaka N, Hassan M, Hibberd P, Lozano JM, Jeena P, et al Oral amoxicillin versus injectable penicillin for severe pneumonia in children aged 3 to 59 months: a randomised multicentre equivalency study. Lancet. 2004;364: 1141–1148. 15451221 10.1016/S0140-6736(04)17100-6

[19] Agweyu A, Gathara D, Oliwa J, Muinga N, Edwards T, Allen E, et al Oral Amoxicillin Versus Benzyl Penicillin for Severe Pneumonia Among Kenyan Children: A Pragmatic Randomized Controlled Noninferiority Trial. Clin Infect Dis. 2014;60(8): 1216–1224. 10.1093/cid/ciu1166 25550349 PMC4370168

[20] Atkinson M, Lakhanpaul M, Smyth A, Vyas H, Weston V, Sithole J, et al Comparison of oral amoxicillin and intravenous benzyl penicillin for community acquired pneumonia in children (PIVOT trial): a multicentre pragmatic randomised controlled equivalence trial. Thorax. 2007;62: 1102–1106. 17567657 10.1136/thx.2006.074906 PMC2094276

[21] Siegel RE, Halpern NA, Almenoff PL, Lee A, Cashin R, Greene JG. A prospective randomized study of inpatient iv. antibiotics for community-acquired pneumonia. The optimal duration of therapy. Chest. 1996;110: 965–971. 8874253 10.1378/chest.110.4.965

[22] Oosterheert JJ, Bonten MJ, Schneider MM, Buskens E, Lammers JW, Hustinx WM, et al Effectiveness of early switch from intravenous to oral antibiotics in severe community acquired pneumonia: multicentre randomised trial. BMJ. 2006;333: 1193 17090560 10.1136/bmj.38993.560984.BE PMC1693658

[23] Castro-Guardiola A, Viejo-Rodriguez AL, Soler-Simon S, Armengou-Arxe A, Bisbe-Company V, Penarroja-Matutano G, et al Efficacy and safety of oral and early-switch therapy for community-acquired pneumonia: a randomized controlled trial. Am J Med. 2001;111: 367–374. 11583639 10.1016/s0002-9343(01)00868-3

[24] Vogel F, Lode H. The use of oral temafloxacin compared with a parenteral cephalosporin in hospitalized patients with pneumonia. J Antimicrob Chemother. 1991;28 Suppl C: 81–86. 1664833 10.1093/jac/28.suppl_c.81

[25] Strohmeier Y, Hodson EM, Willis NS, Webster AC, Craig JC. Antibiotics for acute pyelonephritis in children. Cochrane Database Syst Rev. 2014;7: CD003772 10.1002/14651858.CD003772.pub4 25066627 PMC10580126

[26] Vidal L, Ben Dor I, Paul M, Eliakim-Raz N, Pokroy E, Soares-Weiser K, et al Oral versus intravenous antibiotic treatment for febrile neutropenia in cancer patients. Cochrane Database Syst Rev. 2013;10: CD003992 10.1002/14651858.CD003992.pub3 24105485 PMC6457615

[27] Heldman AW, Hartert TV, Ray SC, Daoud EG, Kowalski TE, Pompili VJ, et al Oral antibiotic treatment of right-sided staphylococcal endocarditis in injection drug users: prospective randomized comparison with parenteral therapy. Am J Med. 1996;101: 68–76. 8686718 10.1016/s0002-9343(96)00070-8

[28] Conterno LO, Turchi MD. Antibiotics for treating chronic osteomyelitis in adults. Cochrane Database Syst Rev. 2013;9: CD004439 10.1002/14651858.CD004439.pub3 24014191 PMC11322802

[29] Zaoutis T, Localio AR, Leckerman K, Saddlemire S, Bertoch D, Keren R. Prolonged intravenous therapy versus early transition to oral antimicrobial therapy for acute osteomyelitis in children. Pediatrics. 2009;123: 636–642. 10.1542/peds.2008-0596 19171632 PMC3774269

[30] Keren R, Shah SS, Srivastava R, Rangel S, Bendel-Stenzel M, Harik N, et al Comparative Effectiveness of Intravenous vs Oral Antibiotics for Postdischarge Treatment of Acute Osteomyelitis in Children. JAMA Pediatr. 2015;169: 120–128. 10.1001/jamapediatrics.2014.2822 25506733

[31] Group AFNST. Treatment of fast breathing in neonates and young infants with oral amoxicillin compared with penicillin-gentamicin combination: study protocol for a randomized, open-label equivalence trial. Pediatr Infect Dis J 32 2013; Suppl.1: S33–38. 10.1097/INF.0b013e31829ff7eb 23945574 PMC3814938

[32] Nuffield Department of Orthopaedics, Rheumatology and Musculoskeletal Sciences (2015) Oral Versus Intravenous Antibiotics (OVIVA) for Bone and Joint Infection. http://www.ndorms.ox.ac.uk/clinicaltrials.php?trial=oviva.

[33] Fletcher C. First clinical use of penicillin. Br Med J (Clin Res Ed). 1984;289: 1721–1723. 6440620 10.1136/bmj.289.6460.1721 PMC1444782

[34] Brandt AM. No Magic Bullet, A social history of venereal disease in the United States since 1880. Oxford: Oxford University Press, 1985

[35] Finniss DG, Kaptchuk TJ, Miller F, Benedetti F. Biological, clinical, and ethical advances of placebo effects. Lancet. 2010;375: 686–695. 10.1016/S0140-6736(09)61706-2 20171404 PMC2832199

[36] Engel MF, Postma DF, Hulscher ME, Teding van Berkhout F, Emmelot-Vonk MH, Sankatsing S, et al Barriers to an early switch from intravenous to oral antibiotic therapy in hospitalised patients with CAP. Eur Respir J. 2013;41: 123–130. 10.1183/09031936.00029412 22653769

