# Supplementary figures and images for: An Unsupported Preference for Intravenous Antibiotics
Source: PLoS Med. 2015 May 19;12(5):e1001825. doi: 10.1371/journal.pmed.1001825 (PMC4437896; doi:10.1371/journal.pmed.1001825)

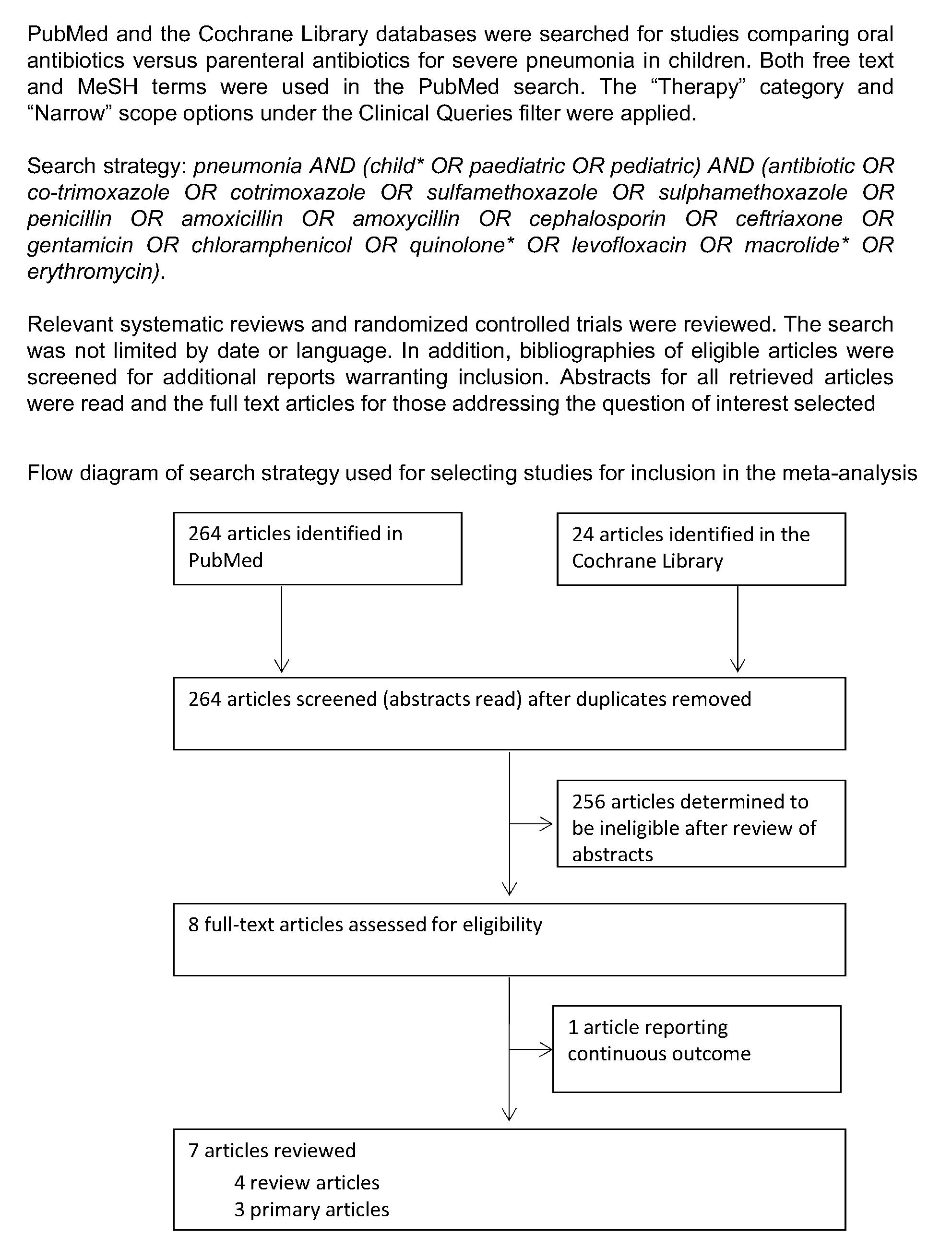

Supplement: S1 Fig — (TIFF) [file pmed.1001825.s001.tiff]
